# The Clinical Potential of 3D-Printed Crowns Reinforced with Zirconia and Glass Silica Microfillers

**DOI:** 10.3390/jfb14050267

**Published:** 2023-05-11

**Authors:** Abdullah Alshamrani, Abdulaziz Alhotan, Ahmed Owais, Ayman Ellakwa

**Affiliations:** 1 Oral Rehabilitation & Dental Biomaterial and Bioengineering, The University of Sydney, Sydney, NSW 2006, Australia; 2 Department of Dental Health, College of Applied Medical Sciences, King Saud University, Riyadh P.O. Box 12372, Saudi Arabia; 3 The X-ray Spectroscopy Node, Sydney Analytical, Core Research Facilities, The University of Sydney, Sydney, NSW 2006, Australia

**Keywords:** 3D-printed dental resins, nanoparticle reinforcement, mechanical properties, translucency

## Abstract

The development of 3D-printed crown resin materials with improved mechanical and physical properties is an area of growing interest in dentistry. This study aimed to develop a 3D-printed crown resin material modified with zirconia glass (ZG) and glass silica (GS) microfillers to enhance overall mechanical and physical properties. A total of 125 specimens were created and divided into five groups: control unmodified resin, 5% either ZG or GS reinforced 3D-printed resin, and 10% either ZG or GS reinforced 3D-printed resin. The fracture resistance, surface roughness, and translucency parameter were measured, and fractured crowns were studied under a scanning electron microscope. The results showed that 3D-printed parts that were strengthened with ZG and GS microfillers demonstrated comparable mechanical performance to unmodified crown resin but resulted in greater surface roughness, and only the group that contained 5% ZG showed an increase in translucency. However, it should be noted that increased surface roughness may impact the aesthetics of the crowns, and further optimisation of microfillers concentrations may be necessary. These findings suggest that the newly developed dental-based resins that incorporate microfillers could be suitable for clinical applications, but further studies are necessary to optimise the nanoparticle concentrations and investigate their long-term clinical outcomes.

## 1. Introduction

The development in dental technology has a positive effect that increases the durability of dental restorations such as crowns and bridges [1]. It also increases the efficiency and the dimension accuracy of these restorations when these technologies have been employed [2,3]. Temporary crowns are crucial for creating permanent dental prostheses due to their ability to protect pulpal and periodontal tissues, prevent teeth from shifting, and sustain occlusal function [4].

These materials meet specific mechanical and biological requirements to be used prior to clinical applications and daily practice [5]. These requirements include withstanding the mastication force, being biologically compatible in contact with oral tissue, securing the bond between the material and tooth structure, and having a desired esthetic outcome [5,6]. Marginal adaptation plays a critical role in the effectiveness and longevity of restorative dentistry. The health of the existing teeth and periodontium can be compromised by a build-up of plaque and microleakage of bacteria engendered by marginal discrepancy [3,7].

Computer-aided design/computer-aided manufacturing (CAD/CAM) and 3D printing are innovative and rapidly evolving technologies that are gaining popularity in the dental industry that provide greater accuracy, reproducibility, speed, and cost-effectiveness [3,8]. Future advances in digital dentistry may provide a solution. Although 3D dental printing is now a common practice, it mainly focuses on prototype manufacturing, such as the fabrication of surgical implant templates, custom impression trays, and cranial models. Interim prostheses have also been manufactured using 3D printing, and the technique is supported by numerous evaluative studies [9,10,11]. Research has also been undertaken into the restoration of primary teeth using digitally printed resin crowns [12,13]. However, due to a lack of suitable printing materials, there is limited research on the use of additive manufacture in definitive restoration. The digital manufacture of effective and durable interim and definitive prostheses relies on the future development of implant materials that are safe, biocompatible, and resistant to corrosion and fracturing [8,14].

Moreover, 3D printing technology can revolutionise the way dental restorations are created. Traditional methods of creating dental restorations, such as using moulds and physical models, can be time consuming and error prone. However, with 3D printing, digital models of the teeth can be quickly and easily created, allowing for more precise and accurate restorations to be printed. This can lead to better fitting and longer-lasting restorations.

Furthermore, the utilisation of 3D printing in dental restoration procedures can facilitate the adoption of novel materials that were not possible with traditional methods. For example, some 3D printing techniques can print restorations with unique properties such as increased strength, flexibility, or biocompatibility. This can lead to the development of new types of restorations that can be used in a broader range of dental procedures. It is worth mentioning that this technology is still under development, and in some cases, it is not yet used in clinical practice. However, many laboratory and clinical trials are being conducted to study the efficiency, strength, and durability of 3D-printed dental materials, as well as their safety and biocompatibility [15,16]. The final product’s strength can also be impacted by the type of material used in 3D printing. Although some materials, such as metals and ceramics, might be stronger than plastics, they are more complex to print and might need specialised tools and procedures [17,18]. Therefore, different materials have been used to reinforce the dental-based composite, such as metal, fibres, and various oxides (aluminium, zirconium, and titanium), resulting in positive and negative outcomes. This is done to enhance the performance of provisional dental resins, which includes improvement of longevity and biocompatibility [14,19,20]. Many of these most recent initiatives have concentrated on improving the properties of nanocomposites by adding nano-fillers and particles to the resin matrix [21]. The inclusion of microfillers or pre-polymerised resin fillers into the resin matrix of micro-filled materials was one method to increase the filler content and, consequently, mechanical characteristics [14,20,22].

Furthermore, research has shown that zirconia and silica have high biocompatibility, meaning they are well-tolerated by the human body and do not cause adverse reactions when used in dental restoration [23,24]. They are also considered appropriate for dental purposes that involve interacting with oral tissues. That are also favoured for their ease of use in 3D printing processes. For example, zirconia can be synthesised into a suspension and used in direct inkjet printing to create dental prostheses with high strength and fracture toughness [24]. Silica can be integrated into dental resin materials used in 3D printing of dental restorations to enhance their mechanical properties.

Numerous studies have been conducted to examine how ceramic nanoparticles impact the characteristics of resin dental composites. One important aspect of dental composites is their chemical stability over time. One study found that the addition of ZrO_2_, TiO_2_, and SiO_2_ nanoparticles to dental composites resulted in increased chemical stability, as evidenced by reduced water sorption and improved mechanical properties [25]. It also found that adding ceramic nanoparticles can improve the chemical stability of dental composites [26,27], while certain additives may have negative effects on stability, leading to reduced mechanical properties due to hydrolysis and degradation of the resin matrix [28]. Zirconium nanoparticles have been suggested to improve the antibacterial efficiency of dental composites according to previous studies [29,30,31], although their use may pose health risks as nanoparticles can penetrate the body and affect organ systems [32]. The toxicity of dental materials is a critical factor for successful clinical use, which depends on their physicochemical properties and biological and toxicological reliability [23,33].

Superior mechanical properties and acceptable aesthetics are required for dental crowns when it comes to temporary dental restorations; it is essential to know the resistance to fracture, microhardness, surface roughness, and aesthetic outcome of the materials used. These properties are significant to consider when the patient will wear the temporary restoration for an extended period, or if they exhibit parafunctional habits such as bruxism (teeth grinding) or clenching. This is especially relevant when planning to fabricate more extensive prostheses, such as fixed bridges. Adding nanoparticles and its effect on various aspects of 3D resin material on mechanical and optical properties, including translucency, have yet to be thoroughly investigated in the literature. The durability and microhardness properties of the materials used in temporary restorations will significantly affect their longevity and the success of future permanent restorations [34]. The demand for the use of 3D printing resin temporarily has recently increased [35]. However, there are limited studies that have assessed the mechanical and optical properties of 3D-printed materials for full dental crowns.

Therefore, further understanding these restorations’ durability is essential for their clinical acceptance. Therefore, the aim of this study was to evaluate the fracture resistance, surface roughness, and translucency of 3D-printed crowns after their reinforcing with silica and zirconia glass microfillers using digital light processing (DLP) technology. The null hypothesis was that there was no effect on the fracture resistance, surface roughness, or translucency parameters of 3D-printed material modified with glass silica and zirconia glass microfillers.

## 2. Materials and Methods

### 2.1. Sample Preparation

The 3D-printed reinforced composites were obtained with the incorporation of silane-coated glass fillers (ultrafine GM35429), with an average particle size of approximately 1.5µm, and silane-coated zirconia particles (ultra-fine G018307), with an average particle size of approximately 0.4 µm (Schott, Landshut, Germany), at different concentrations of 5% and 10% (*w*/*w*). The microfillers were added to the printable resin solutions and stirred for 24 h using a magnetic stirrer. The mixture was then sonicated for 45 min in a water bath. A detailed description of the materials used in this study is provided in Table 1

**Figure 1:**
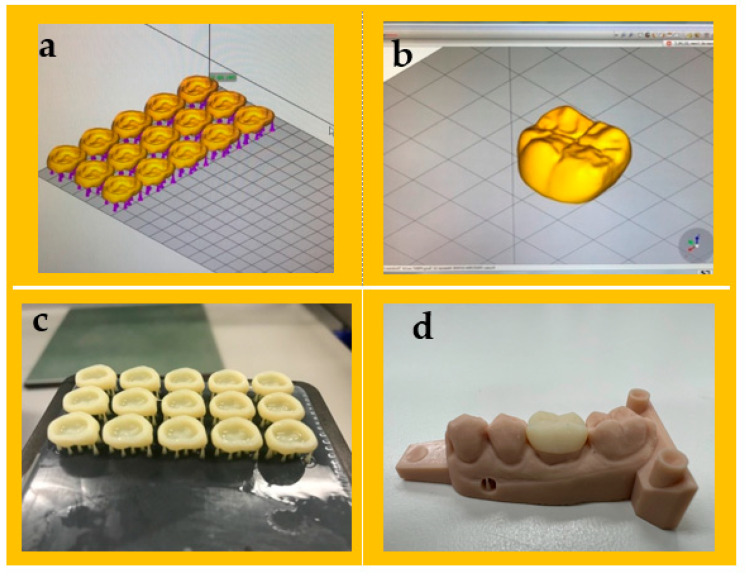
Design of dental crowns and 3D printing workflow. (**a**) Setup of dental crowns and generation of support structures using 3D printer software. (**b**) Final design of the full crown. (**c**) The 3D-printed crown on the printing platform. (**d**) Final placement of the printed crown on the model.

To create a model for the study, a maxillary resin typodont (AG-3 ZPVK, Frasaco GmbH, Tettnang, Germany) was utilised. The typodont had a single-unit first molar. The typodont was then scanned using a desktop optical scanner (E1 scanner, 3Shape, København, Denmark), and the scanned model was produced using a 3D printer (Asiga MAX™; ASIGA, Sydney, Australia) using model resin materials (Asiga DentaMODEL, Asiga, Sydney, Australia). The stereoscopic images from a scanner were used to virtually design the planned prosthesis. The resin crowns with uniform thickness of 0.5 mm from the occlusal, buccal, lingual, and proximal surfaces were designed using Exocad software (Exocad GmbH, Darmstadt, Germany) in three dimensions, according to ISO 4949-2019 (as depicted in Figure 1 [36]. After that, the data were converted to standard tessellation language (STL) files and transmitted to 3D printer software program to slice the virtual designs and determine the printing parameters. The sample size for the current study was determined by considering the findings and recommendations from previous studies [37,38].

**Figure 2:**
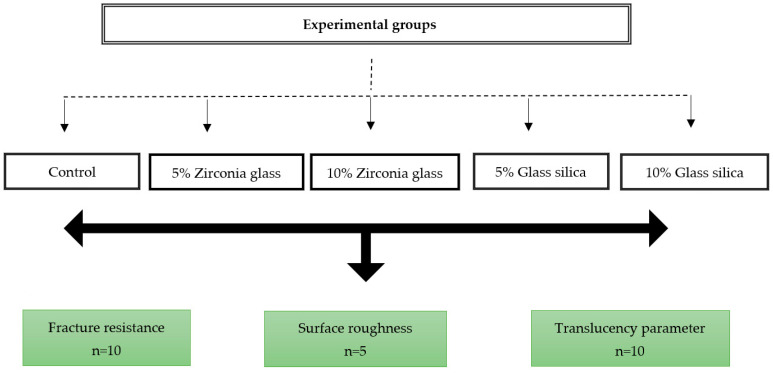
Flowchart of the overall experimental process of this study and experimental types.

A total of 10 virtual crown designs for each group were positioned on the build platform of the 3D printer. A layer thickness of 50 μm and a 0-degree print orientation were determined. Samples were then fabricated using a DLP printer (Asiga MAX™; ASIGA, Sydney, Australia). Full contour restoration (*n* = 50) with a minimum of 1 mm occlusal thickness, 0.8 mm axial thickness, and 50 µm cement space near the margins of the crowns and 100 μm for the rest of the internal space were designed using CAD software, as illustrated in Figure 1. The disc-shaped sample dimensions (10 × 2.0 mm) with a minimum of 2 mm thickness were used to evaluate surface roughness and translucency parameter following specification the American Dental Association (ADA) [39]. Following the printing process, the samples were subjected to an ultrasonic cleaning and sonicated at 35 kHz in a water bath (Transonic 460/H, Elma, Germany) for 5 min. The purpose of this step was to eliminate any excess resin monomers. The 3D-printed crowns were post-cured for 10 min in a post-curing unit (Solidilite V, Shofu Dental GmbH, Ratingen, Germany). For luting the crowns, the G-Cem luting cement was used (GC Corporation, Tokyo, Japan). The G-Cem cement was activated and mixed in a capsule mixer for 10 s. The elongated tip was used to apply the cement to the inner surface of the prepared crown, which was then immediately inserted and held under pressure for 10 s. Excess cement was removed with a brush, and then the crown was light-cured for 20 s. The five groups of current study were carried out as follows: control (*n* = 25); 3D-printed reinforced with the following: 5 wt% glass filler (*n* = 25), 5 wt% zirconia glass (*n* = 25), 10 wt% glass filler (*n* = 25), and 10 wt% zirconia glass (*n* = 25). Each group was further divided on the basis of the testing method: fracture resistance (*n* = 10 per group), translucency parameter (*n* = 10 per group), and surface roughness (*n* = 5 per group), which were tested for each group, as shown in Figure 2.

### 2.2. Fracture Resistance

**Figure 3:**
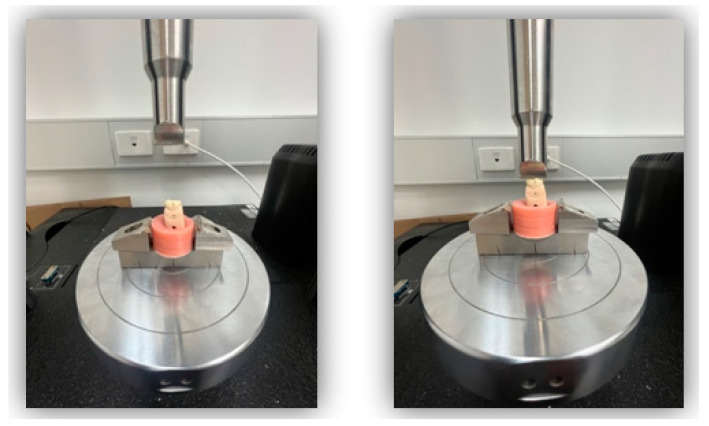
Fracture resistance testing assembly in universal testing machine.

Fracture resistance of ten 3D-printed crowns per group (*n* = 10) was performed according to ISO 4949-2019 [36] with a universal mechanical testing machine (Instron 5965; Instron, Canton, MA, USA). An investigation was undertaken to analyse the fracture resistance of a sample group of 3D-printed resin crowns. In preparation, 24 h before commencing, each crown was immersed in distilled water and kept at a temperature of 37 °C to simulate the typical oral temperature. The test aimed to reproduce the uniaxial force typically exerted by the occlusion between upper and lower teeth. A single cycle of compressive force was applied via a 4 mm diameter steel bar impacting the midline fissure of the molar crown (Figure 3). This exerted a crosshead speed of 1 mm/min and was repeated until the tooth fractured. The test utilised a dedicated classification system designed by Ellakwa et al. [40] to record the requisite force (*n*) and mode of fracture (Table 2).

### 2.3. Surface Roughness

This study utilised non-contact (AC/tapping mode) atomic force microscopy (AFM; Bruker Innova, Billerica, MA, USA) to calculate the surface roughness (Ra) of the resin crowns was measured according to ISO 25178 [41]. The surface was scanned at 0.5 Hz using an industry-standard Tap300AI-G probe, acquired from Budget Sensors. This resonated at an average frequency of 300 kHz and applied an average force constant of 40 n/m to generate images with a resolution of 256 × 256 pixels. Three different image sizes were exported for each specimen crown: 10 × 10 µm^2^, 25 × 25 µm^2^, and 45 × 45 µm^2^. These were then analysed using Bruker NanoScope Analysis data-processing software (Version 1.9, Bruker AXS, Karlsruhe, Germany)

### 2.4. Color Change Analysis (Translucency Parameters)

A 44 mm wide target mask and an automated variable sample illumination were combined with a spectrophotometer (LabScan XE, Hunter Associates Laboratory Inc., Reston, VA, USA) to measure the reflectance values for ten specimens from each tested group. The spectrophotometer was calibrated on the basis of the manufacturer’s instructions before testing a black trap and a white tile. The saturation level and hue dimensions are represented by two chromatic axes at right angles to one another: a* (red-green parameter difference) and b* (yellow-blue parameter difference). Value or lightness is represented by the third axis, L*, parallel to the chromatic planes. Each specimen was evaluated six times in total using the translucency parameter (TP): three times each against a black background and three times against a white background. The following equation is used to calculate the TP value of a material [42]:

TP = [(L*B − L*W)^2^ + (a*B − a*W)^2^ + (b*B − b*W)^2^] ^½^ (1)

where L* refers to the lightness, a* from redness to greenness, and b* from yellowness to blueness, and they are measured against black (B) and white (W) backgrounds.

### 2.5. Fractographic Analysis

A scanning electron microscope (SEM) (TM3000, Hitachi High Technology, Tokyo, Japan) was used to conduct fractographic analysis on fractured specimens of 3D-printed teeth crowns. The surfaces of the fractured crowns were coated with gold-palladium alloys using a sputter coater and examined at an accelerating voltage of 20 kV. Additionally, the surface structure of the fractured crowns at high magnification (×2000) was also examined. The fractographic analysis was conducted in accordance with the guidelines provided by the Academy of Dental Materials (ADM) [43].

### 2.6. Statistical Analysis

The data were analysed and presented as mean values with corresponding standard deviations (SD). The normality of the data was assessed by examining their distribution and conducting the Kolmogorov–Smirnov test. One-way analysis of variance (ANOVA) was used to evaluate the effect of added microfillers on fracture resistance, surface roughness, and colour change values. The significance level was set at *p* ≤ 0.05 and a 95% confidence interval. Statistical analysis was performed using GraphPad PRISM software (GraphPad Software Inc., San Diego, CA, USA).

## 3. Results

### 3.1. Fracture Resistance

**Figure 4:**
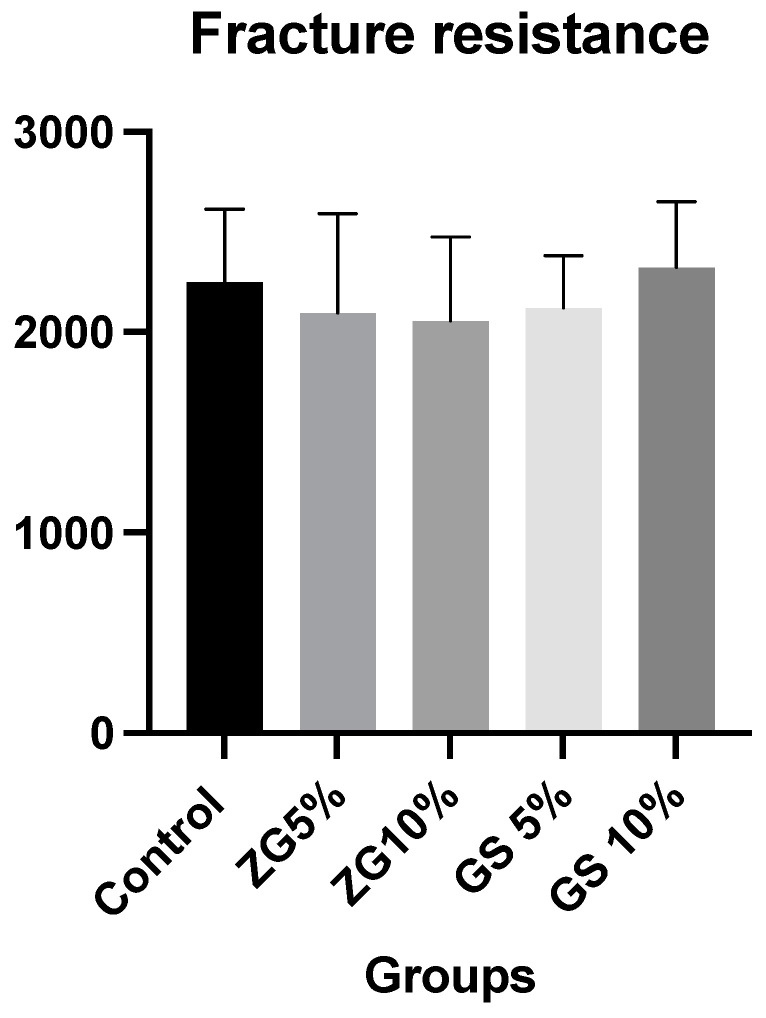
Bar chart showing the mean and standard deviation (SD) of fracture resistance values for all tested groups.

The means and standard deviations of the fracture resistance results of all tested experimental groups were as follows: control group: 2250 ± 364.20 N; ZG 5%: 2095 ± 495.70 N; ZG 10%: 2055 ± 420.1 N; GS 5%:2121 ± 260.10 N; GS10: 2325 ± (325.8). Mean and standard deviation values of fracture resistance in different groups are presented in Figure 4 and Table 3. The mode and number of failures observed in each group are displayed in Table 4. Among all groups, the most common mode of failure was a crown fracture through the midline, with half of the crown displaced or lost (48%). Additionally, the second most frequent type of failure was mode II, which corresponded to the loss of less than half of the crown (38%). The other modes of failure had fewer failures overall. There were no significant differences between loads at break between the groups, regardless of the reinforcement condition (F (4.45) = 0.88, *p* = 0.481). However, it was slightly higher for 10% glass silica groups (2325 ± 325.80) than the other groups.

### 3.2. Surface Roughness

**Figure 5:**
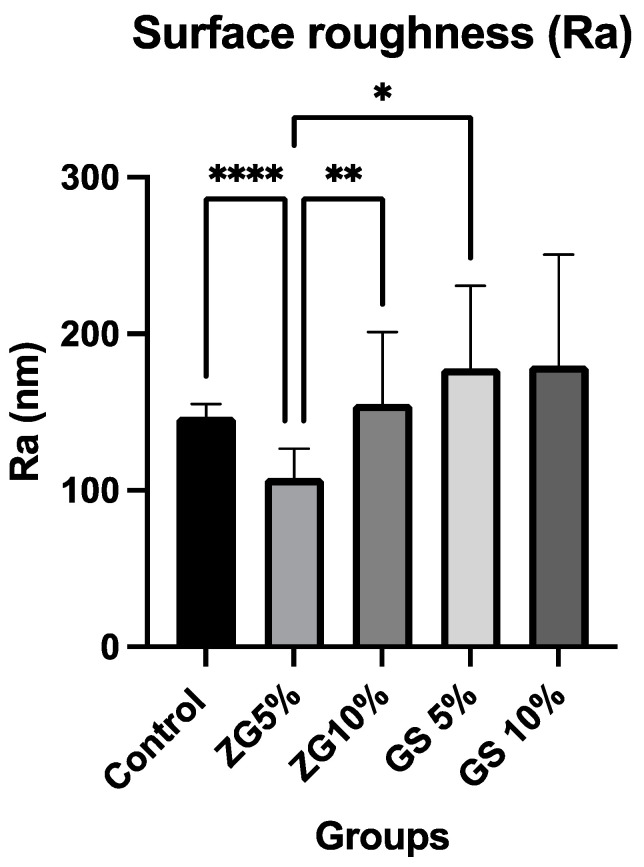
Bar chart showing the mean and standard deviation (SD) of surface roughness (Ra, nm) values for all tested groups. “*” indicates the significance level of *p* < 0.05, “**” represents the significance level of *p* < 0.01, and “****” denotes the significance level of *p* < 0.00001 between the groups.

**Figure 6:**
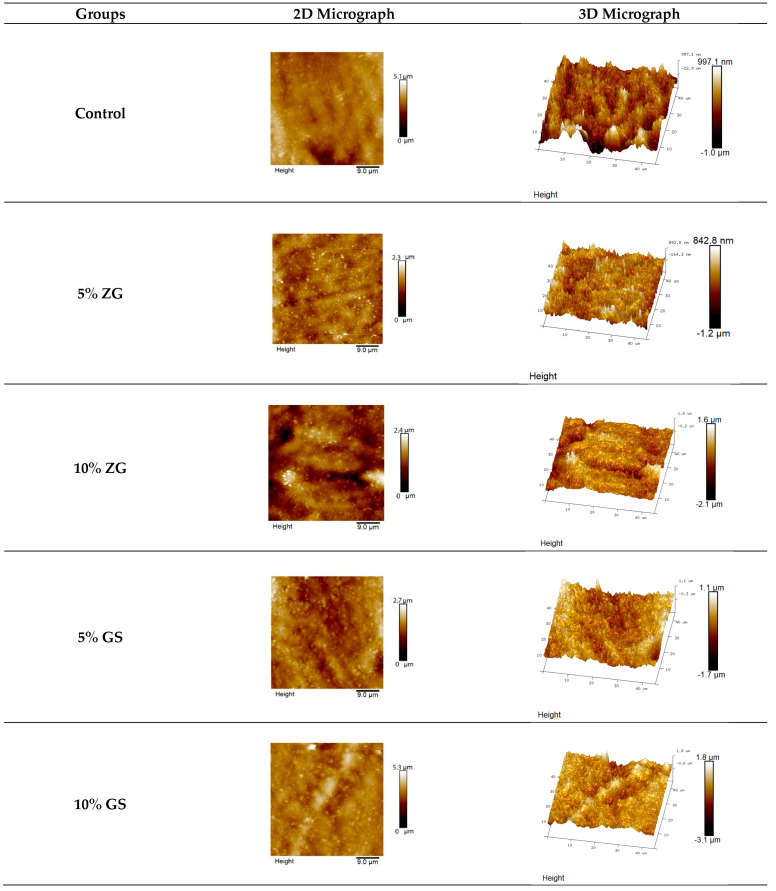
AFM micrographs: comparing surface roughness of 3D-printed specimens with nanoparticle fillers (zirconia and glass silica) at varying concentrations. ZG5% had the lowest roughness, GS10% had the highest. Reinforced groups showed higher roughness, except ZG5%. No significant difference was found between GS5% and GS10%.

The results for the mean and standard deviation of average roughness (Ra) values are shown in Table 3 and Figure 5. One-way ANOVA tests showed significant differences among groups tested within each material (*p* < 0.0064). Tukey tests for multiple comparisons between the groups revealed that some of the groups led to a statistically significant difference. AFM topographic micrographs of the 3D-printed specimens, representing the groups of microfillers (zirconia glass and glass silica) incorporated with different concentrations (5% and 10% *w*/*w*), in addition to the control group, are illustrated in Figure 6. The AFM topographic micrographs are presented in two-dimensional (2D) and three-dimensional (3D) formats. GS10% groups had the highest average roughness values among all tested 3D-printed resins at 180 ± 75 (*p* ≤ 0.05), and ZG5% had the lowest average roughness value: 108 ± 20 nm. Except for ZG5% (108 nm), all groups that were reinforced had higher surface roughness values compared to the control groups, which consisted of ZG10% (155 nm), GS5% (178 nm), and GS10% (180 nm). There was no significant difference found between the GS5% and GS10% groups (*p* > 0.05).

### 3.3. Translucency Parameter

**Figure 7:**
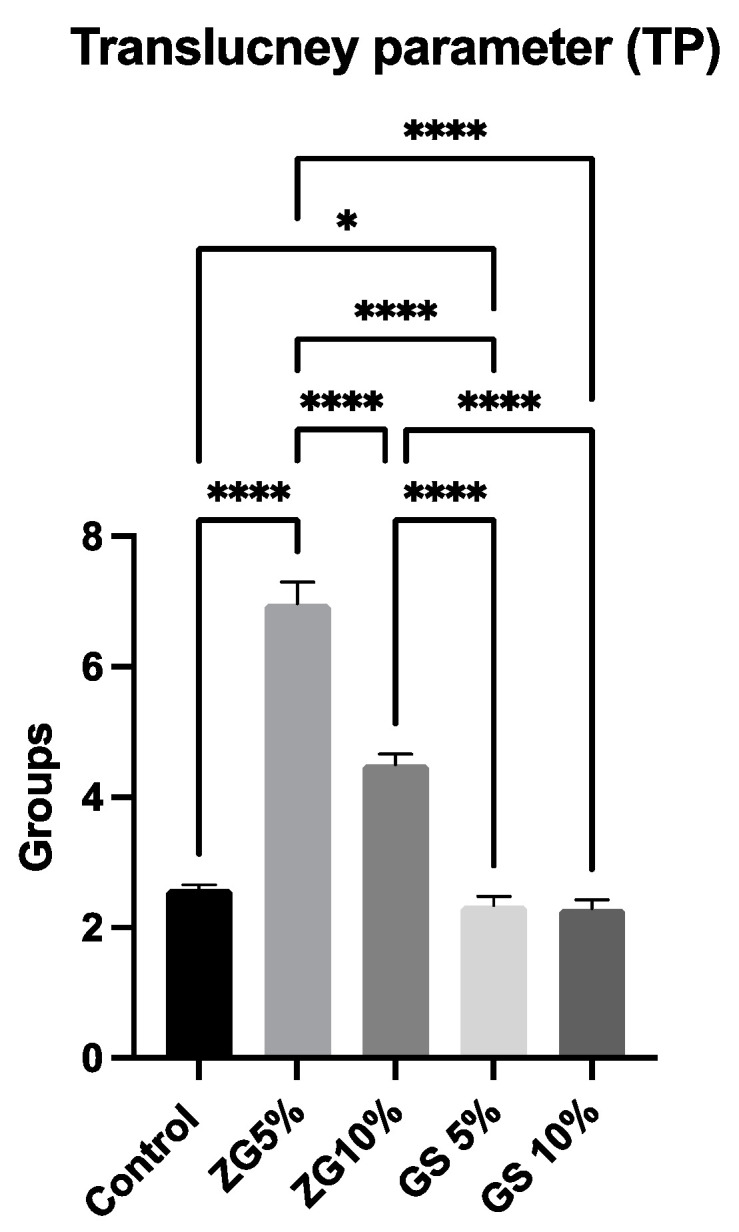
Mean and SD of translucency parameters (TPs) of all tested groups. “*” indicates the significance level of *p* < 0.05, and “****” denotes the significance level of *p* < 0.00001 between the groups.

**Figure 8:**
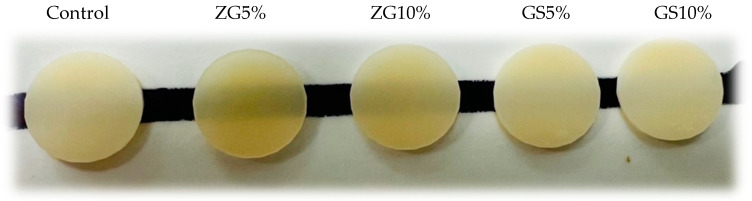
Specimens (2 mm thick) of (left to right) control, ZG5%, ZG10%, GS5%, and GS10%.

The TP results of all 3D-printed dental materials are shown in Table 3 and Figure 7. One-way ANOVA tests showed significant differences among the groups tested, regardless of the reinforcement condition (F (4.45) = 1168, *p* ≤ 0.001). Tukey tests for multiple comparison between the groups revealed that all the groups were statistically significant from each other (*p* ≤ 0.05). Translucency was found to be highest in the ZG5% group (6.965 ± 0.33) (*p* < 0.001). The translucency values of the tested groups were compared, and it was found that 5%GS (2.338 ± 0.13) and 10%GS (2.293 ± 0.12) had the lowest values among the 3D-printed resins (*p* < 0.001), as opposed to the other tested groups. Representative specimens were photographed (Figure 8).

### 3.4. SEM Analysis

**Figure 9:**
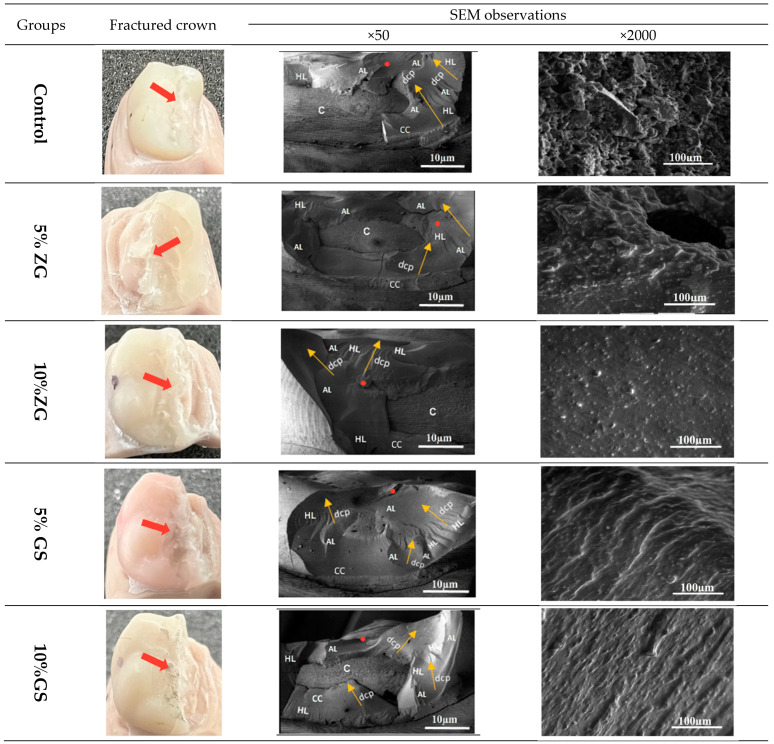
Representative photographic and SEM images of fractured crowns. The images on the left are photographic images. On the right are two SEM images with magnifications of ×50 and ×2000. SEM of ×50 illustrated the fracture site with the following: (c: core; AL: arrest line; HL: hackle line; red dot: origin of fracture; dcp with yellow arrows: direction of crack propagation).

Figure 9 depicts representative SEM images of the tested materials for fractographic analysis. The topographies of all the fractured crowns are shown for each experimental group. All crowns tested had catastrophic adhesive failures, exposing either the cement layer or the dental crown to unrestorable fractures. However, no abutments were found to be damaged. The fracture site was explained on SEM images with the following features: arrest lines, hackle lines, origin of fracture, and direction of crack propagation.

## 4. Discussion

Three dimensionally printed resins have grown in popularity among clinicians because of their ability to reduce design times, expedite fabrication, and improve performance. However, several factors can influence the final material properties as with resin composites. These include particle size, shape, monomer type, and inherent properties of the materials and polymerisation equipment, such as polymerisation time, light source, and post-processing steps. In recent years, there has been a growing trend towards utilising pre-fabricated zirconia crowns to aesthetically restore primary teeth [44]. Nevertheless, available zirconia crowns in the present market have some size and customisation limitations, making 3D printing a new option for primary tooth restoration. In addition, 3D-printed resin crowns can be custom-made for each patient, unlike other prefabricated crowns.

The present study investigated the influence of material reinforcement using glass silica or zirconia glass, with different weight percentages of 5% and 10% on the mechanical and physical preparties of printed crowns. These crowns were investigated for three different properties: fracture resistance, surface roughness, and translucency. It was hypothesised that reinforcement conditions have an influence on fracture resistance, surface roughness, and translucency parameter properties. Except for the fracture resistance property, all the tested properties of 3D-printed crowns showed significant findings. Nevertheless, the outcome presented that 3D-printed crowns showed comparable results between tested groups regarding fracture resistance. The highest surface roughness was observed at 5% and 10% glass silica 3D-printed crowns. Hence, the null hypothesis was partially accepted. Although in vitro studies cannot entirely replicate clinical situations, they can offer guidance to clinicians when using the tested materials. The fracture resistance values of the tested materials did not differ significantly. The fracture loads endured by the materials tested in this study were significantly higher than the reported maximum biting forces in the posterior region (500–900 N) [45]. The fracture load of the tested groups ranged from 2055 to 2325 N.

Two important findings deserving further discussion emerged from this study: the results indicated a clinical equivalence between the fracture resistance of the different 3D-printed test specimens, and the mean value of the forces required to fracture the sample crowns was shown to exceed 2000 N. The latter finding aligns with previous research into the fracture resistance of 3D-printed reinforced resin crowns: forces between 1495.05 and 2303.7 N were recorded for tests on teeth located in the back of the mouth [12,46]. This is significant as it exceeds the average human bite force, which has been shown to increase with age: humans aged 6–8 years exert a force of 78 N rising to a maximum of 176 N at ages 18–20 years [47].

When assessing the fracture resistance of dental materials, it is essential to consider the entire study design, including the characteristics of the test material, the abutment design and its material properties, and the parameters of the fracture loading [48,49,50]. Furthermore, the cement layer and its thickness can affect the values for maximum occlusal force on the crowns [51,52,53]. Therefore, the results obtained from in vitro studies cannot be translated to clinical situations at once because the load-to-failure testing method only provides information under extreme conditions, and in-depth investigation is necessary.

Various experimental conditions can yield different results for the fracture force of tested materials [54,55]. The elasticity modulus of the supporting structure is a crucial factor in fracture properties, as has been previously demonstrated [54,56]. A previous investigation showed that the fracture strength of dental crowns increases with a higher E-modulus of the supporting material [57]. In this study, the die made with 3D/DLP technology had an E-modulus of 2.1 GPa, while the resin cement had an E-modulus of 8.7 GPa. However, human dentine has an E-modulus ranging from 7 to 16 GPa [58]. The study setup may tend to favour composite materials due to the low elastic modulus of the DLP fabricated abutment. Therefore, it might be preferable to use human teeth or materials with a comparable E-modulus for in vitro fracture tests. Multiple factors can impact the surface roughness of a dental restoration, including the method of fabrication, oral conditions, occlusal loading, dietary habits, and the composition of the materials used [14,59]. The physical and chemical attributes of dental resin play a pivotal role in determining the longevity of dental restorations. For instance, surface roughness is critical to consider when producing dental restorations. The surface roughness of dental restorations is essential for their longevity and aesthetic appeal. Smoother surfaces, according to research, are tougher and less prone to complications such as superficial staining, reduced gloss appearance, and diminished surface hardness [60,61]. Rougher surfaces are also linked to gingival inflammation, secondary caries, and bacterial plaque accumulation [62,63]. The current study assessed surface roughness on the basis of reinforcement conations (glass silica or zirconia glass) with different weight percentages of 5% and 10%. GS10% groups had the highest roughness values among all the tested 3D-printed resins, while ZG5% had the lowest.

The outcomes of our investigation indicated a statistically significant variance in surface roughness among the test groups concerning their reinforcement conditions. Regardless of the added weight percentages, the 3D images exhibited a smoother surface for the control group relative to the ZG and GS groups. These findings suggest that dissimilar material composition is crucial in determining surface smoothness, a phenomenon well documented in prior research [64]. Specifically, the unmodified group, which consists of methacrylate material containing a smaller filler, is believed to generate a smoother surface that is easily polishable to achieve a smooth finish [65,66]. Therefore, in order to achieve better results in the current study, it might be recommended to implement a plan that involves decreasing the size of the fillers and using advanced mixing techniques, which can successfully minimise the roughness of the surface.

Their translucency significantly influences the selection of metal-free materials. To assess this characteristic, a material’s colour variation is measured when placed against a standard black and white background at a specific thickness, using the CIELab* system. Johnston (2014) proposed this method to assess translucency [67] directly. However, since the material’s translucency is thickness dependent, and no ISO standard is available for evaluating translucency in dentistry, a 2 mm thickness was used in the current study to compare the findings with those reported in previous research. A wide range of translucency values was observed among the materials analysed. The ZG 5% group exhibited the highest translucency value of 6.965 ± 0.33 in this investigation, while the 5% GS and 10% GS groups demonstrated the lowest translucency values of 2.338 ± 0.13 and 2.293 ± 0.12, respectively, compared to other groups tested. Increased opacity levels were observed when there was a significant difference in refractive index between the polymeric matrix and the reinforcing filler or opacifying compounds. This effect is attributed to reflections and refractions occurring at the boundary between the matrix phase [68]. Therefore, the increased TP value in the 5% ZG group can be ascribed to integrating zirconia microfillers into a heavily cross-linked 3D resin matrix. The zirconia microfillers’ smaller diameter than the visible light wavelength leads to minimal light scattering and enhanced light transmission, resulting in increased translucency [69]. A reported value of 6.85 has been documented for TP in human dentin specimens with a thickness of 2 mm [70]. Despite variations in the experimental conditions, the TP values observed in the 5% ZG groups were found to be similar to those of natural human teeth. The translucency of dental materials is influenced by the scattering of light, which is affected by a disparity in refractive indices between the filler and matrix of dental resins [71]. According to a study by Ota et al., a robust association was observed between the refractive index and TP values of dental resins [72]. Additionally, the TP value showed a reduction with an increase in filler content when the size of the filler remained the same.

The inclusion of fillers in printable resins can result in increased viscosity, which may have a detrimental effect on printability, causing issues such as clogging, uneven flow, and reduced printing accuracy [73]. Furthermore, fillers have the tendency to settle over time, leading to uneven distribution within the resin and inconsistent mechanical properties in printed objects [74]. These factors could potentially explain why there was no positive improvement observed in terms of fracture resistance results. The current investigation is limited by its reliance on in vitro methodology. Such an approach must be revised to replicate the diverse chemical and mechanical properties within the oral cavity. Hence, it is imperative to undertake further optimisation measures to ensure the efficient dispersion of microfillers into the resins. However, the variation in fabrication methods prevents a direct comparison of our results with previous findings. Although crown-shaped specimens were used in this study, it is suggested that further investigations should be conducted in intraoral environments.

## 5. Conclusions

Within the limitation of the current study, the addition of ZG microfillers did not impact the fracture resistance of 3D-printed crowns, as all added concentrations produced similar results to unmodified crown-based resin. However, incorporating ZG and GS increased the groups’ surface roughness values. In addition, 5% ZG microfillers enhanced the translucency parameter of 3D-printed resin. The findings indicate that adding zirconia glass and glass silica microfillers into the 3D-printed resin matrix may offer a beneficial option to unmodified 3D-printed resin and can be considered for dental applications.

## Figures and Tables

**Table 1:** Material details used in the current study.

Material	Type	Manufacturer
Everes Temporary (dental resin)	3D-printed resin	Sisma, Italy
Glass fillers (ultrafine GM35429)	Additive particles	Shofu Inc., Kyoto, Japan
Zirconia glass (ultrafine GM018-307)	Additive particles	Shofu Inc., Kyoto, Japan
G-cement capsule A2	Cement resin	GC corporation, Tokyo, Japan

**Table 2:** Classification of modes of failure modified from [40].

Code#	Description
I	Minimal fracture capable of refinishing and rapier
II	Less than half of crown lost
III	Crown fracture through midline, half of crown displaced or lost
IV	More than half of crown lost
V	Sever fracture of the crown

**Table 3:** The means and standard deviations of the fracture resistance (FR), roughness average (Ra) values, and translucency parameter (TP) values that were found for the 3D-printed material.

Group	Fracture Resistance	Roughness Average (Ra)	Translucency Parameter (TP)
Control	2250 (364.20) ^a^	147 (±9) ^a^	2.590 (0.06) ^a^
5% ZG	2095(495.70) ^a^	108 (±20) ^b^	6.965 (0.33) ^b^
10% ZG	2055 (420.0) ^a^	155 (±49) ^ac^	4.502 (0.15) ^c^
5% GS	2121(260.10) ^a^	178 (±56) ^ac^	2.338 (0.13) ^d^
10% GS	2325(325.8) ^a^	180 (±75) ^a^	2.293 (0.12) ^d^

ZG: zirconia glass; GS: glass silica. Same superscripted lowercase letters indicate groups not statistically significantly different when compared by Tukey multiple comparisons post hoc analysis (*p* > 0.05).

**Table 4:** The mode of failure that was reported for each experimental group.

Groups	Mode of Failure				
	I	II	III	IV	V
Control	1	1	8	0	0
5% ZG	0	5	4	1	0
10% ZG	0	3	3	4	0
5% GS	0	5	5	0	0
10% GS	0	5	4	1	0
Total	1	19	24	6	0

## Data Availability

The data presented in this study are available on request from the corresponding author due to privacy.
